# Notes of a Trip to New York, Philadelphia and Richmond

**Published:** 1904-05

**Authors:** James McFadden Gaston

**Affiliations:** Atlanta, Ga.


ATLANTA

### Journal-Record of Medicine.

Successor to Atlanta Medical and Surgical Journal, Established 1855, and Southern Medical Record, Established 1870.

Vol. VI. MAY, 1904. No. 2.

BERNARD WOLFF, M.D., EDITOR, Nos. 319-20 Prudential.

Published Monthly.

M. B. HUTCHINS, M.D., BUSINESS MANAGER, 1007-1008 Century Bldg.

### ORIGINAL COMMUNICATIONS.

### NOTES OF A TRIP TO NEW YORK, PHILADELPHIA AND RICHMOND.

By JAMES McFADDEN GASTON, M.D.
Atlanta, Ga.

It is always a difficult matter for a physician who has been doing a general practice for ten years to leave his practice and take a course in any of the hospitals or schools of medicine and surgery, yet it is very desirable that each practitioner should see what is going on in other centers, where he can study and observe without having responsibility of the cases that he sees; and, having received a prize given by the *International Journal of Surgery*, entitling the writer to a course in the New York School of Clinical Medicine, it was an opportunity which should be embraced. It was also made pleasant by the kindness of friends and relatives in New York and Brooklyn as well as in Virginia.

My wife and I started on a trip of this kind on Thursday, February 4th, and reached Richmond first, on the 5th.

Here, it may be said, that many doctors of reputation, at home and in the South, make an excellent place for study. It is the seat of two medical colleges and several hospitals of importance. I saw Dr. Stuart McGuire, son of Dr. Hunter McGuire, and visited his hospital, where I had the pleasure of seeing him operate. His technic is very simple and his results are satisfactory from every point of view, for a young man who has had a large surgical practice. As a friend of Dr. McGuire, I was glad to see him show the same skill in surgery that his father possessed. I did not see into the hospital until later, as Dr. McGuire was absent when I first called.

In New York the opportunities for clinical study are equal to any in the world. There are probably one hundred hospitals in Greater New York. Of these we visited one dozen of the most important. One of the smaller hospitals is St. Marks, but it has the reputation in Europe, as well as in this country, for being the theatre of many brilliant operations, and also many excellent results from them.

Here, Dr. Carl Beck, a German physician, who has been in New York for twenty years, is the president and moving spirit, but any physician can take his patient there, provided there is room. I also saw some of the most important operations in every portion of the body. There were two operations for cancer of the tongue and several mastoid operations, and operations upon the chest, empyema, as well as operations upon the neck, involving the complete excision of the internal jugular vein and many cervical glands. Dr. Beck is one of the authorities on the subject of the X-ray, having written a book which is now ready for publication, and he gave a practical demonstration of the utility of this means of diagnosis of an osseous cyst in the shoulder, being an epiphyseal formation, found in younger patients. This was a young lady, sixteen years of age, who had been treated for rheumatism and other things unsuccessfully. When Dr. Beck saw her, he had an X-ray examination made and the skiagraph showed that there was a cyst of the head of the bone, yet, upon inspection, there was very little to be noted in difference of the two shoulders. The patient, however, could not raise her arm to any distance above her shoulder. When the shoulder was examined more particularly by myself, at the instance of Dr. Beck, there was noted an elasticity of the shoulder, which was in striking contrast to the hardness of the other shoulder. Before the operation I had an opportunity of seeing this patient several times. The operation was also witnessed by me and confirmed the diagnosis completely. The patient will, no doubt, be relieved by the method of treatment adopted. It consisted in the cutting of the shoulder and evacuating this cyst, which was in the substance of the bone, but did not seem to be hard to reach, as the knife alone was sufficient to get into it from the outside. A curette was used to scrape out the contents of the cyst, which was jelly-like and partly like marrow, and then the cavity was packed with gauze.

Another case, in which a bone was involved, and also illustrating the X-ray, was that of a fracture of the neck of the femur. The patient had been treated for three or four weeks with the ordinary splints, but there was a shortening of several inches. It was necessary to cut down into the joint and thoroughly disengage the fragments in the intertrochanteric line, and in this there was drilled a hole in which was placed an ordinary steel screw that is used by carpenters. It was made to pass through the head of the bone and into the upper fragment, which was in the acetabulum. This screw was left for a short time, until union could take place, and then was to be removed. The union had not existed before on account of a fibrous mass lying between the fragments. The X-ray had shown that there was a displacement of the bone at the head of the femur and gave rise to the diagnosis. The complete correction of this displacement was very marked, after the operation, there being no shortening, and a complete cure might be expected under all circumstances. The treatment of these fractures is always difficult, and if a surgical operation, here described, corrects the deformity, the problem is solved in the treatment of fractures of the neck of the femur, involving the capsule. The X-ray should be used in order to ascertain this fact early in the treatment of these cases, and should be a guide in the performance of an operation.

The method of wiring here does not avail anything, because the wire can not be placed in the upper fragment, which lies very close in the socket, and only the end of the screw can penetrate the place.

I have gone into detail in description of these two cases, where the X-ray was beneficial in diagnosis, and because they probably show better than anything else the thorough method of the treatment by Dr. Beck in these cases.

He had several operations for the removal of the appendix, one for excision of the lower jaw, and the operations were all very instructive. They did not present the striking features that the above did. He uses a great deal of iodoform and seems not to have had any bad results from it. One operation for hernia which he performed had some points of difference and difficulty that are not usually encountered, and was in a case of a man who weighed 250 pounds and who had a recurrent rupture. The muscles were used in a funnel shape by a special method that prevented the escape of the contents of the abdomen. It was very much like the Bassimi operation in many respects. Kocher, in his Surgery, mentions Dr. Beck's form of transplanting the rectus muscle in the treatment of hernia as originated with Dr. Beck, though Bloodgood, of Baltimore, has also claimed to be the first one to use it.

Another hospital of interest is the King's County Hospital of Brooklyn. Here we have two well-known surgeons and anatomists, who operate on Thursdays. It is an institution containing a great many charity patients, and for that reason the clinical material is more varied than in private hospitals. Dr. A. T. Bristow, Clinical Professor of Surgery in the Long Island Medical College, is the attending surgeon, and Dr. W. G. Campbell, Professor of Anatomy in the same college, is his assistant. They usually operate together, but at times in a rush of work not only does Dr. Campbell take the whole responsibility of the operation, but sometimes they have a house surgeon who is given an opportunity to practice what he has seen and to perform the operation himself.

Dr. Bristow has performed several operations for excision of the carotid artery for malignant growths of the face. His work during the time I had the pleasure of seeing him operate consisted in operating for hernia and one for the removal of the glands of the neck. He also performed an operation for the transplantation of the facial nerve which was paralyzed and required to be united with the spinal accessory nerve.

He operated upon one of the house staff for appendicitis, making a gridiron incision, and then having a purse-string of catgut to enclose the stump of the appendix. I was well acquainted with the young man, who was proposing to go out as a medical missionary. The case was one of recurrent appendicitis, and need not necessarily have given him alarm but for the fact that he was afraid another attack would be when he was too far away from medical skill to be treated successfully by a surgeon. The appendix was hardened and cord-like and there was already an effusion of lymph into the peritoneal cavity, so that there was danger of another and most serious onset within a month. Dr. Bristow was perfectly satisfied with the operation, thinking it had been performed as successfully as he was capable of doing it.

This feeling that a surgeon has of satisfaction with his own work is the best reward as well as the best test of his surgery. It is not easy to please one's self in a duty where an ideal to be reached is a high one.

Dr. Moses went on the table with the same Christian fortitude that sustained him in making a decision for the foreign field, and he was calmer and more resigned than the average patient before an operation. His pulse, as I had occasion to note upon my visit to him before the operation, was only 60 and strong and regular. The nurses in the hospital where he had worked, and in which he had seen others suffer, were very sympathetic and kind to the house surgeon, but one of his patients was more demonstrative in his feelings than any one else connected with the hospital. He was a little Italian boy named Jake, who had been himself under the knife for an appendicectomy, and on account of an abscess that existed needed to be dressed frequently. I saw his wound dressed and felt the pain he must suffer was intense. He was up and able to be about the room the next time in the hospital, however; and, going into the room of Dr. Moses, he looked somewhat serious, and the house physician remarked to him, "Well, Jake, you can punch me next time and get even." Instead of taking this as an opportunity for retalliation, Jake broke down in tears, and the doctor was so surprised and overcome himself that he ordered the boy out. After awhile Jake came back and saw the doctor being wheeled off into the anesthetic room. Another boy called out, "Jake is crying for you." The next time I saw Jake he was in smiles, for the doctor had been through the operation successfully, and a day or two had passed and no serious trouble seemed to be going to come of it. Such are the lights and shades of a house surgeon's experience. [N. B.—Later news from Brooklyn : Dr. Moses recovered entirely.]

Dr. Beck used an ordinary ligature, and after ligating and cutting off the appendix and stripping back the mesoappendix, he took an ordinary stitch from one side to the other of the stump and included this portion of mesentery into the stitch, so as to cover the stump.

Dr. George Ryerson Fowler, of Brooklyn Hospital, performed an operation in which he used the thermocautery for the stump of the appendix and had a purse-string connected with the cuff of the serous coat of the appendix, and pushed it back so as to be buried, but not entirely inverted into the cecum.

Dr. Fowler has written a book upon appendicitis, and his work is very interesting in all departments of surgery. I saw him do an operation of the gall-bladder, in which there was no stone in the gall-bladder, but there was a stone in the common bile-duct. The method of drainage was the most interesting feature of this operation, for it consisted in the insertion of a tube into the gall-bladder and stitching the edges of the gall-bladder a little above the point where the tube was finally to rest, enabling the tube to be pushed down against some purse-string sutures that had been placed below them. The tube was long enough to be placed in a basin on the floor near the bed, and secured drainage without any leakage.

There was also in the practice of Dr. Campbell, at St. John's Hospital in Brooklyn, a patient who had been operated on for a stone in the common bile-duct. In this case the stone was embedded so as to require an incision of the common bile-duct with its removal. The patient was doing very well the next time I saw her.

Dr. Campbell had an interesting operation at the Bushwick Hospital. It was a gastrostomy for cancer at the cardiac end of the stomach, and the patient had not eaten a square meal for a year or more, I suppose, and had not been able to swallow anything for some days. There was need of doing the operation as quickly as possible, and by actual time it required seventeen minutes. At the end of this time a tube had been inserted into the stomach through an artificial esophagus, and a glass of milk and whiskey was poured into a funnel at one end of the tube and went into the stomach, and this was the way she would be fed.

There had been a case of the chief of police in New York, who was fed altogether by this means and lived and performed his duties some years after the operation.

Dr. Campbell is interested in the treatment of cancer by electricity, and he gave this case a trial of a method of electrolysis and cataphoresis which I described to him, and also gave a personal demonstration of its method of application for his benefit. He has a case of recurrent carcinoma of the breast in which he desires to use this.

It is fitting to say in this connection that both Dr. Fowler and Dr. Campbell promised to make use of the means we have adopted for treating inoperable cases of cancer.

Dr. Fowler has three hospitals in which he can use this, and Dr. Campbell has also ample opportunity to test it. It is to be hoped that the same uniformly successful results may be found by them as we have already met with, and as Dr. Fraser, of Thamesville, Ontario, has also found.

In direct connection with this may be mentioned a visit I paid to Dr. G. Betton Massey, of Philadelphia, who has been treating cases of carcinoma with massive cataphoresis. He gave me some of his electrodes, which are now made of amalgamated zinc, which he has found better than the gold formerly used by him.

He will soon issue a revised edition of his book, "Conservative Gynecology," which promises to be an advance upon anything that has been done in the line of electrotherapeutics. His views now are in a line with the chemical process that takes place by the current when a medicine is applied on the positive or negative pole of the battery. I suggested to him the use of Donovan's solution upon his electrodes in place of pure mercury, and I think he will give it a trial. It is much more diffusible and requires less current than his process.

There is a book called "The Outlines of Electro-Chemistry," by Professor Jones, of Johns Hopkins University, which may be studied in connection with Dr. Massey's work, as he recommends it very highly.

The behavior of chemicals under a weak galvanic current is a very instructive field of study, and offers to the chemist, as well as the physician, many important suggestions for treatment, which may be embraced and generally utilized by the profession under the general head of "Cataphoric Medication." It is more effective even than hypodermic medication and will be more and more serviceable to mankind, as the medicines can be made to enter the system in a direct line with the part affected, and the doses of the drugs become better known and their effects described.

I gave a day or more to the study of the offices of the doctors who use electricity almost exclusively in their practice. Dr. Wm. J. Morton, who has for many years been prominent in the treatment of diseases by advanced therapeutics, and especially electricity, is one of the scientists applying practically his knowledge to treat his patients. He prefers the use of radium to some of his former methods of treatment. He has written a book on "Cataphoresis," but it has not been so widely read as it should have been. He showed me some of his instruments for this process of introducing medicines through the skin, which I think are still superior to anything heretofore accomplished with radio-active water or other means of using this new principle of fluorescence in the body; however, it is attracting some attention and we shall look forward to his experiment with interest. The process will be hard to understand, and yet it is a very simple one. It consists in giving to the patient a few doses of the bisulphate of quinine, until the tissues of the body are thereby rendered capable of either reflecting or refracting the rays given out by radio-active water, which is also taken into the stomach. The idea of such an infinitesimal dose producing great results is itself somewhat of a barrier to its general acceptauce by the profession.

The study of radium has not gone sufficiently far to demonstrate what it is and what we are to expect from it. It is now receiving considerable attention from the specialists in stomach troubles, as a means of determining whether the stomach is in position or not. Lincoln's light is a term that I heard used to qualify a little electric-incandescent lamp that is swallowed by patients who have been subjected to treatment similar to that described as being used by Dr. Morton, and serves to light up the walls of the stomach that are already fluorescent when seen in the dark.

There are other means of producing this fluorescence besides radium. They are by means of uranium, thurium, fluorescin and other substances of like character, reminding one more of the "Will o' the wisp" than of anything else, and I venture to predict will be equally transitory.

The physicians in London have already discarded radium in the treatment of cancer. It is not probable that radium will be found any more serviceable than the X-ray in the treatment of cancer.

The work of Mrs. Margaret A. Cleaves is also studied, and she considers that radio-active water will lose its power within six or eight hours at the most. She has a very good apparatus for giving light baths and using the Finsen light, which she claims to have had in use a good many years before Finsen put it in practice himself. She has shown in her writings that this process has given good results in the treatment of some cases.

Dr. William Benham Snow is devoting himself to methods of advanced therapeutics, and had quite a number of patients under treatment by the X-ray and static machine, his vacuum tubes for the use of the high-frequency current and the vibrator. The vibrator is one of the most interesting instruments that I have seen. It depends upon principles of communicating its vibrations to the skin through a rotary wheel. It is used in massage and exercises imparted to the muscles similar to that gained by their contraction with electricity. It should be found beneficial in headache and nervous diseases, and is very pleasant and easily taken.

While we are speaking of the results of light, heat and electricity, it may be well to remark that the surgeons are using these agents more now than formerly, and that Dr. John A. Wyeth has adopted the injection of boiling water into the tissues, so as to get the coagulating effect. I saw him apply the injection in a case of angioma. He has been using it in a great variety of cases, and I was glad to see a demonstration of his method. There is an ordinary metal syringe like that used in hypodermic injection, only with a larger barrel and needle. The water is hot enough even to scald, as shown by throwing it up in the air and letting it come down on the hands of those who would witness it. It is a ready method, one that can be utilized by the general practitioner in cases of aneurism or nevus or any other collection of blood, such as hemotoma.

Dr. John A. Wyeth is a Southern man, and has just returned from a trip to Florida. He is generally well known in New York and Brooklyn.

I attended the New York Surgical Society meeting and heard a paper by Dr. Lillienthal, describing upwards of forty cases of cholecystectomy. He has had good results in excision of the gall-bladder and the discussion of his paper led to many interesting points that were brought out by the surgeons in regard to this operation. This paper will doubtless be read with discussion, and I can only say that the impression left upon me was that the gall-bladder should be removed in many cases where it is left. It is a useless organ after its walls are diseased, and in health it does not hold a great amount of bile, but probably serves as an overflow reservoir. When it is obstructed and the outlet no longer serves to allow the bile either to flow in or flow out, it acts as a foreign body or tumor and may give rise to malignant disease or indigestion.

Whether it may be compared to the appendix is another question. We can very well try to avoid extremes in both gall-bladder surgery, as in other departments of gastro-intestinal surgery. It is not necessary to remove the gall-bladder just because another operation is performed in the region near it, nor is it well to resort to appendicectomy just because we are aware that it gives trouble sometimes, and because the surgeon may be operating upon the ovaries or the kidneys.

I saw two interesting operations upon the urinary bladder—one by Dr. Ryerson Fowler, of Brooklyn, N. Y., and the other by Dr. Carl Beck, of New York. One was a case of suprapubic lithotomy, and was an Italian boy about sixteen years of age. He had come into the Brooklyn Hospital for treatment, and gave symptoms of tenesmus and pain upon urinating, together with other symptoms of calculus, yet he had not been sounded for stone until they placed him upon the operating-table. The reason for this was that he was examined by the house surgeon, who found the urethra was so small as not to admit of the instrument without pain, and he waited until he was under the anesthetic. He was pretty sure of his diagnosis, and when an ordinary steel sound was inserted into the bladder the calculus was plainly felt. I was permitted to feel the instrument and note the grating of its beak against the stone. Then the bladder was filled with a solution of boracic acid, and an attempt was made to get the click of the instrument against it, but this was not so distinct as at times; still there was no doubt of the existence of the stone, and the operation was done for its removal. Neither was a rectal bag used, nor was the bladder filled with fluid more than to wash it out.

Dr. Fowler explained that the rectal bag was dangerous in causing rupture of the rectum, and that he had not used it for some years. He also considers it unnecessary to extend the bladder so as to carry the peritoneum above and out of the way of the operator, for he claims that the surgeon should be able to reach the part of the bladder without this help. I told him that would do for a man as he was, but it would not do to teach the average surgeon, because it was difficult enough to reach the bladder with a guide like we had and was now well recognized as being of value. He performed the operation successfully, where another might fail.

This led to the discussion of the urethra, and, to my surprise, he stated he never used a guide in performing operations for external perineal urethrotomy. His experience with this is similar to that of Dr. W. S. Goldsmith, of our city, who has adopted the treatment of these cases without a guide. It requires a knowledge of the appearance of the structure, which even the anatomist may find to differ after a diseased condition, while the pathologist understands how entirely changed the special features due to a great many false passages are often made by instruments in examining the urethra.

Dr. Fowler claims that these very false passages are troublesome in introducing a guide, and that the surgeon may be led wrong by this process, while he can not stray far off if he has the whole field open and before him.

This is begging the question, because we know that we have to operate without a guide when we have certain conditions that prevent the use of the guide, whether from stricture that is impermeable or one that is permeable but can not be found.

I have, myself, found a great deal of trouble in reaching the bladder by the perineal route on account of this false passage. In one case which I operated upon under cocaine the stone in the bladder could be touched at first by the grooved guide, but later had escaped into the tissues, so that it could not be found. Probably an operation without a guide would have been equally unsatisfactory, for the guide was not probably in the bladder at all, but in a false passage.

The other case of subrapubic cystotomy was for a papilloma, and was done at St. Marks Hospital soon after I reached New York. It was a transverse incision about an inch from the pubic bone, and when the bladder was reached was incised so as to enable the operator to touch the papilloma with the thermocautery. It was done long enough before I left for me to see the good effect of the operation.

*202 Prudential Building.*

